# Recurrent Ameloblastoma: Clinical Manifestation and Disease-Free Survival Rate

**DOI:** 10.1155/2022/2148086

**Published:** 2022-08-09

**Authors:** Andrii Hresko, Roman Palyvoda, Olha Burtyn, Yurii Chepurnyi, Andrii Kopchak, Marco Helder, Tymour Forouzanfar

**Affiliations:** ^1^Department of Maxilla-facial Surgery and Innovative Dentistry, O.O. Bogomolets National Medical University, 13, T. Shevchenko Blvd, Kyiv 01601, Ukraine; ^2^Department of Head and Neck Tumors, National Cancer Institute, Kyiv, Ukraine; ^3^Department of Oral and Maxillofacial Surgery, Oral Pathology, VU University Medical Center, Academic Center for Dentistry Amsterdam (ACTA), Amsterdam, Netherlands

## Abstract

**Objectives:**

Ameloblastoma is a slow-growing epithelial odontogenic neoplasm of the jaws with a high recurrence rate. The main treatment strategies for this lesion are radical or conservative surgical approaches. The aim of the present study was to analyze clinical presentations, histological types, and treatment strategies of recurrent ameloblastoma and to define its disease-free survival (DFS) rate.

**Materials and Methods:**

Twenty-four cases of recurrent ameloblastomas, treated between January 2009 and July 2021, were enrolled in this study. Medical files from each patient, including gender, age, size of the lesion, localization, patient complaints, clinical manifestation, radiographic appearance, histological type, surgical management, and treatment results were reviewed and analyzed retrospectively.

**Result:**

Out of 69 operated primary ameloblastomas, the rate of recurrence was 35%. Out of 24 recurrent cases, 21 developed after conservative treatment and 3 after radical treatment. In most cases, recurrences were found in the mandible (*n* = 20). A unilocular pattern was predominant in radiographic examination (44%). Estimated 3-year DFS was 84.5 ± 4.8%, and the 5-year and 10-year DFS were 73.0 ± 6.3% and 43.9 ± 8.343.9 ± 8.3%, respectively.

**Conclusion:**

Results obtained in the present retrospective study proved the necessity of long-term follow-up after both conservative and radical treatment approaches. The DFS median in our study was 8 years (95% CI 6 years–10 years). For recurrent cases, radical resection with histologically free margins after exact MRI determination of the ameloblastoma border within the soft tissues should be considered as the method of choice to avoid secondary recurrence.

## 1. Introduction

Ameloblastoma is a slow-growing benign odontogenic neoplasm that is locally aggressive and has a high recurrence rate [1]. Ameloblastoma has no gender predilection, but it is predominant in patients between 30 and 60 years old [2]. The mandible is affected much more frequently than the maxilla (88 vs. 12%) [3]. According to the 2017 World Health Organization (WHO) classification of head and neck tumors, ameloblastoma can be divided into three subtypes: conventional ameloblastoma (solid/multicystic variant), unicystic ameloblastoma, and extraosseous/peripheral types [4]. Conventional ameloblastoma was recognized as the most common type and is associated with significantly higher recurrence rates [2, 5, 6]. Primary treatment of ameloblastoma is surgical and can be classified into conservative and radical [7]. Conservative methods such as enucleation or extended curettage are less invasive and require less operation time, but these methods are associated with significantly higher recurrence rates and the need for secondary reoperations [8]. Radical surgery, including marginal resection, segmental resection, hemimandibulectomy, and maxillectomy, is associated with lower recurrence rates but often results in serious aesthetic and functional impairment, decreasing the patient's quality of life, and often requires complex reconstructive surgery [9]. With conservative treatment, 55–90% of cases recur, whereas, the recurrence rate with radical treatment is 15–25% [10, 11]. In both cases, the treatment prognosis depends on the ameloblastoma type, clinical and radiological signs, and histologically confirmed diagnosis [12]. Unicystic and extraosseous ameloblastoma, according to Hertog et al., can be treated conservatively with adequate success rates, while the more aggressive conventional ameloblastoma requires radical treatment in most cases [13]. Ameloblastomas that recur after initial conservative or radical surgery are a major challenge for the surgeons as their clinical presentations and topographic relations with the surrounding anatomic structures may be distorted, and more radical interventions are needed for a positive long-term prognosis [14]. The reccurences can appear in the remained parts of affected jaws, in the soft tissues, or in the transplanted bone, used for defect replacement. The recurrences can be detected within 1 to 10 years after primary surgery [15]. Recurrent ameloblastomas that manifested 15 to 30 years after the initial surgery were also reported [16]. Most of the cases analyzed in the literature present the solid type of *t* ameloblastoma (particularly the follicular variant) [17]. Possible predisposal for recurrence after surgical treatment is the histologic type, location of the lesion, and its penetration into the soft tissues via a destroyed cortical layer [18]. The surgical strategy, manifestation, and prognosis for recurrent ameloblastoma are discussed in numerous scientific publications [19–21]. Most reports present a very small series or single clinical cases, with a marked variety of clinical symptoms and individual characteristics, making the conclusions unconvincing [22]. The aim of the present study was to analyze clinical presentations, histological types, and treatment strategies of recurrent ameloblastoma and to define its disease-free survival (DFS) rate.

## 2. Materials and Methods

Twenty-four cases of recurrent ameloblastoma, treated at the Kyiv regional clinical hospital and the National Cancer Institute (Kyiv, Ukraine) from 1 January 2009 to 31 July 2021, were retrospectively analyzed. These cases were set aside for further analysis from the cohort of 69 ameloblastoma patients with not less than three years of follow-up. The study was approved by the Bioethics Committee of Bogomolets National Medical University, Kyiv, Ukraine (protocol no. 107). The inclusion criteria were the following: histologically confirmed diagnosis of recurrent ameloblastoma with definite surgical treatment performed; well-documented cases with clinical, radiological, and histological data. For each patient, the data concerning gender, age, personal history (alcohol, smoking, and drug addiction), size of the lesion, localization, patient complaints, clinical manifestation, radiographic appearance, surgical management, histological type, and complications were collected from medical records reviewed and analyzed retrospectively. All recurrent ameloblastoma cases were histologically confirmed and classified according to 2017 WHO classification [4]. If a mixture of patterns was observed in single ameloblastoma, the predominant pattern was considered for subclassification. In all cases, CT with 3-dimensional visualization was applied and carefully reviewed.

All patients enrolled in the study underwent conservative or radical surgical treatment depending on the size, location of the lesion, its clinical and radiological manifestations, and histological diagnosis. Conservative treatment included enucleation and extended bone curettage, while radical treatment consisted of bone resection (segmental or marginal) and hemimandiblectomy according to the recommendations of Hendra et al. [9].

Analysis of the results of the study was performed using the statistical software EZR *v*. 1.54 (graphical user interface for R statistical software version 4.0.3, R Foundation for Statistical Computing, Vienna, Austria) [23]. The mean value (*Х*) and standard error (*m*) were calculated for the quantitative data. Frequency (%) was calculated for the qualitative data. The survival analysis (disease-free survival rate) was performed by the Kaplan—Meier method. Risk ratios (HRs) with 95% confidence intervals (95% CI) were calculated for risk of disease recurrence analysis. To evaluate the effect of risk factors on disease-free survival (the calculation of adjusted HR), a Cox proportional hazard regression model was utilized. A stepwise method was used to select the independent factors of the multivariate models. A *P* value less than 0.05 was considered statistically significant.

## 3. Results

**Figure 1:**
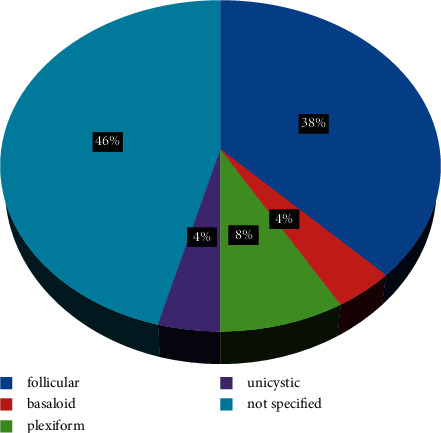
Distribution of the recurrent ameloblastoma histological subtypes.

Within the cohort of 69 primary ameloblastoma in our study, the frequency of recurrence was 35%. In some patients, the recurrences develop even after secondary surgical interventions: Out of 24 patients with recurrent ameloblastoma, 35 episodes of recurrence were diagnosed (mean 1.45 ± 0.88 per patient). The recurrent ameloblastoma was presented mostly by conventional (solid/multicystic) ameloblastoma (*n* = 23) and slightly less frequently by unicystic ameloblastoma (*n* = 1) (*p*=0.657). The following histological subtypes were identified as follows: 9 follicular (37.5%), 1 basaloid (4.1%), 2 plexiform (8.3%), 1 unicystic (4.1%), and 11 not specified cases (45.8%) (Figure 1). Twenty cases of recurrent ameloblastoma were detected in the mandible. The most frequent location for recurrence development in the mandible was the body (62.5%), then the angle (37.5%), and the ramus (33.3%). In 8 cases, recurrences developed when the primary tumor affected more than one anatomical area of the mandibular. In the maxilla, all recurrences were located at the premolar or molar region. The radiographic examination detected 16 cases of unilocular and eight multilocular patterns with dimensions ranging from 2 to 10.5 cm. There was a small male predominance among the patients 54% male vs. 46% female. The average age of the patients with recurrent cases was 41.08 ± 12.66 years, ranging from 15 to 62 years.

The performed multifactorial analysis showed the absence of any kind of correlations between the clinical features, histological type of ameloblastoma as well, the applied treatment method, and the probability of recurrence in the evaluated cohort of patients.

**Figure 2:**
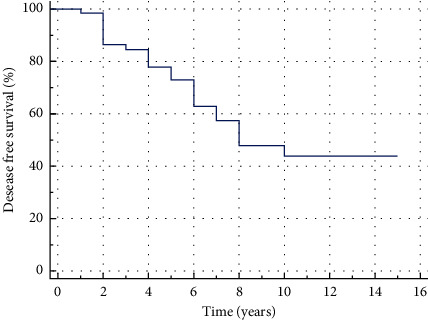
Kaplan–Meier disease-free survival curve for the ameloblastoma patients.

**Figure 3:**
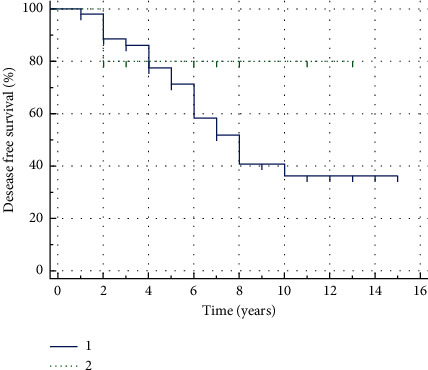
Kaplan–Meier disease-free survival curves for the patients with different types of treatment: conservative treatment = NoR (1) and radical treatment = R (2).

The DFS median in our study was 8 years (95% CI 6 years–10 years). Estimated 3-year DFS was 84.5 ± 4.8%, the 5-year DFS and 10-year DFS were 73.0 ± 6.3% and 43.9 ± 8.343.9 ± 8.3%, respectively (Figure 2). However, the difference between DFS curves, defined for each group separately, in favor of radical treatment was not statistically significant in this cohort of patients (*p*=0.172) (Table 1). The DFS median for conservatively treated patients was 8 years (95% CI 6 years–10 years). The DFS median for surgically treated patients was not reached in 13-year observation period (Figure 3).

The radical treatment of recurrent ameloblastoma was applied to 14 patients and included the extended bone (and, if necessary, soft tissue) resection with a 1.5 cm clear margin around the radiologically determined borders. Secondary recurrence after such treatment was noted in four cases. In three of them, the secondary recurrence developed in the soft tissues of the infratemporal fossa with no connection to the bone. In one case , episodes of recurrence in soft tissues were noted over the observation period of 7 years, resulting in multiple surgeries. The other ten patients were treated conservatively. Secondary recurrence in these patients developed in four cases.

## 4. Discussion

Ameloblastoma is a benign neoplasm with aggressive behavior, local invasiveness, and a high recurrence rate [22]. Recurrent ameloblastoma is always a major challenge for the maxillofacial surgeon, as it requires additional extended resection in distorted topographic conditions. Such cases are usually associated with significant aesthetic and functional deficiency and require multistage reconstructive operations. In most cases, recurrent ameloblastoma is associated with incorrect treatment tactics and failure of first surgery [24–32]. Last meta-analyses demonstrated that the incidents of recurrence in ameloblastoma treatment are within the range of 15% to 29% [1, 6, 33, 34]. Laborde reported a higher recurrence rate of 40% [19]. The present study analyzed 69 patients treated over 13-year period. The recurrence rate after treatment of primary ameloblastoma was 35%. We did not find any statistically significant correlation between the risk of recurrence development and gender, age, type of ameloblastoma, location, radiological, or histopathological. The same results were presented in Almeida et al., systematic review and meta‐analysis [6]. The pooled recurrence rate for solid ameloblastoma was 4.7% after radical and 32.8% after conservative treatment (*n* = 21). For unicystic ameloblastoma, these numbers were 25% after conservative treatment and no recurrence after radical surgeries. These findings show that the solid or multicystic type behavior is slightly more aggressive than the unicystic type and, however, was not statistically significant in this study (*p*=0.330). In our series, the conventional (solid) ameloblastoma was observed in 23of 24 of the recurrent cases, the follicular type being predominant (37.5%) for this group. The last finding is in agreement with data presented by Hong et al. (46%), Hertog et al. (41%), and Fregnani et al. (44%) [2, 6, 34, 35]. The systemic review by Reichart et al. demonstrated that the risk of recurrence correlated significantly with the histopathological type [36]. These findings are the same as in the research of Hong, who also demonstrated a correlation between histopathology and recurrence rate [2]. However, the association between histologic patterns and recurrent ameloblastoma is still controversial. Au et al. found no association between the histological pattern and recurrence rate as well as the other authors came to the same conclusions [37].

According to Muller and Slootweg, most cases of recurrent ameloblastoma are diagnosed within 5 years after surgery [29]. In the earlier series by Olaitan et al., more than 80% of recurrent cases were diagnosed within the first 5 years after surgery, with the longest period before the manifestation of the recurrent ameloblastoma of more than 13 years [38]. In our study, 34% of cases were diagnosed in terms more than 5 years after surgery, with the mean period between surgery and the clinical or radiological manifestation of the recurrence 7.75 ± 1.75 years and the longest being 12 years. The expected DFS median in our study was 8 years (95% CI 6 years–10 years). The analysis of DFS with the use of the Kaplan—Meier method in our cohort of patients demonstrated comparable results of 3-year survival rate with similar studies of Yang et al. [39, 40]. However, 5- and 10-year disease-free results of abovementioned studies differed from those obtained in our cohort of patients. The DFS rate, defined in our study decreased dramatically comparing 5- and 10-year recurrent free results. This suggested that the results were dependent on underestimated conditions because the main clinical and pathological conditions of our cohort were similar to those shown by Yang et al. [39].

According to the literature, recurrence can occur in remote postoperative periods. Adebayo et al., Hayward, Collings, and Harrison have reported recurrence after 21, 30, and 49 years, respectively [41–43]. A possible source of recurrence is the remaining cells at the osteotomy site or the retained soft tissue ameloblastoma islands during the surgical procedures, especially in complex regions like the infratemporal fossa. The morphological study of Carlson and Marx uses radiograph samples in 82 cases after resection to demonstrate that the ameloblastoma histologically extends beyond 2–8 mm, and the average spread of it to the bone is 4.5 mm [21]. It proves the necessity of extended resections of 1.5–2 cm margins around the radiologically determined ameloblastoma borders. Additionally, the worst prognosis in recurrent ameloblastoma is associated with the spread of the lesion outside the bone with the involvement of the soft tissues [17]. Such cases cannot be seen on the CT, and MRI is often required to plan the extent of resection. In the present study, 14% of the recurrences were asymptomatic and revealed by X-ray or CT examination. It proves the importance of long-term radiologic follow-up for early diagnostics and adequate treatment of recurrent cases.

In our study, three recurrent ameloblastomas occurred exceptionally in the soft tissues, after the spread of the primary neoplasm outside the bone. In these 3 cases, the recurrence developed in the soft tissues of the infratemporal fossa with no connection to the bone. However, the frequency of ameloblastoma that recur in the soft tissues after radical treatment can be more significant. In the series of 26 cases of recurrent ameloblastoma reported by Olaitan et al., the tumor spread into the soft tissues in 4 cases (15%) [38]. In a later multicenter analysis by Arotiba et al., 23.3% of 30 cases of recurrent ameloblastoma were located in the soft tissues [44]. Yang et al. defined the perforation of the cortical bone as a risk factor for recurrence [39]. At the same time, Eckardt et al. have reported recurrences in bone grafts after the long-term follow-up [31, 45–48]. In our study, only one recurrence developed in patients with bone grafting procedures after radical jaw resections.

The main limitation of our study was the relatively restricted number of well-documented cases with a predominance of conservatively treated ones. Prevalence of more conservative treatment resulted in preference for less complicated surgery, associated with a low rate of postoperative complications and aesthetic and functional deficiencies that can influence the patient's quality of life. At the same time, the radical approach often requires several complex and expansive surgical procedures with an unclear prognosis.

## 5. Conclusions

Results obtained in the present retrospective study proved the necessity of long-term follow-up after both conservative and radical treatment approaches. The DFS median in our study was 8 years (95% CI 6 years–10 years). For recurrent cases, radical resection with margins should consider the method of choice to avoid secondary recurrence, with MRI determination of the ameloblastoma border within the soft tissues.

## Figures and Tables

**Table 1:** Disease-free survival rate for patients with different types of treatment.

Term	Expected recurrence-free survival rate, % ± *m*%	
	Conservative treatment	Radical treatment
1 year	98.0 ± 2.0	100%
3 years	86.1 ± 5.3	80.0 ± 10.3%
5 years	71.3 ± 7.5	80.0 ± 10.3%
10 years	36.2 ± 9.0	80.0 ± 10.3%

## Data Availability

The data used to support the findings of this study are included in the article.

## References

[1] Hendra F. N., Van Cann E. M., Helder M. N. (2020 Jan). Global incidence and profile of ameloblastoma: a systematic review and meta-analysis. *Oral Diseases*.

[2] Hong J., Yun P. Y., Chung I. H. (2007). Long-term follow up on recurrence of 305 ameloblastoma cases. *International Journal of Oral and Maxillofacial Surgery*.

[3] Milman T., Ying G. S., Pan W., Livolsi V. (2016). Ameloblastoma: 25 Year experience at a single institution. *Head and Neck Pathology*.

[4] Wright J. M., Vered M. (2017). Update from the 4th edition of the World Health organization classification of head and neck tumours: odontogenic and maxillofacial bone tumors. *Head and Neck Pathology*.

[5] Hresko A., Burtyn O., Pavlovskiy L. (2021). Controversies in ameloblastoma management: evaluation of decision making, based on a retrospective analysis. *Medicina Oral, Patología Oral y Cirugía Bucal*.

[6] Almeida R. D. A. C., Andrade E. S. D. S., Barbalho J. C., Vajgel A., Vasconcelos B. C. D. E. (2016). Recurrence rate following treatment for primary multicystic ameloblastoma: Systematic review and meta-analysis. *International Journal of Oral and Maxillofacial Surgery*.

[7] Singh T., Wiesenfeld D., Clement J., Chandu A., Nastri A. (2015). Ameloblastoma: demographic data and treatment outcomes from Melbourne, Australia. *Australian Dental Journal*.

[8] Ghandhi D., Ayoub A. F., Pogrel M. A., MacDonald G., Brocklebank L. M., Moos K. F. (2006). Ameloblastoma: a surgeon’s dilemma. *Journal of Oral and Maxillofacial Surgery*.

[9] Hendra F. N., Natsir Kalla D. S., Van Cann E. M., de Vet H. C. W., Helder M. N., Forouzanfar T. (2019). Radical vs. conservative treatment of intraosseous ameloblastoma: systematic review and meta-analysis. *Oral Diseases*.

[10] Ooi A., Feng J., Tan H. K., Ong Y. S. (2014). Primary treatment of mandibular ameloblastoma with segmental resection and free fibula reconstruction: achieving satisfactory outcomes with low implant-prosthetic rehabilitation uptake. *Journal of Plastic, Reconstructive & Aesthetic Surgery*.

[11] Sammartino G., Zarrelli C., Urciuolo V. (2007). Effectiveness of a new decisional algorithm in managing mandibular ameloblastomas: a 10-years experience. *British Journal of Oral and Maxillofacial Surgery*.

[12] McClary A. C., West R. B., McClary A. C. (2016). Ameloblastoma: a clinical review and trends in management. *European Archives of Oto-Rhino-Laryngology*.

[13] Hertog D., Schulten E. A. J. M., Leemans C. R., Winters H. A. H., Van Der Waal I. (2011). Management of recurrent ameloblastoma of the jaws; A 40-year single institution experience. *Oral Oncol [Internet]*.

[14] Gurol M., Jeff Burkes E. (1995). Peripheral ameloblastoma. *Journal of Periodontology*.

[15] Aramanadka C., Kamath A. T., Kudva A. (2018). Recurrent ameloblastoma: a surgical challenge. *Case Reports in Dentistry*.

[16] Adekeye E. O., Lavery K. M. (1986). Recurrent ameloblastoma of the maxillo-facial region. *Journal of Maxillofacial Surgery*.

[17] Hertog D., van der Waal I. (2010). Ameloblastoma of the jaws: a critical reappraisal based on a 40-years single institution experience. *Oral Oncol [Internet]*.

[18] Becelli R., Carboni A., Cerulli G., Perugini M., Iannetti G. (2002). Mandibular ameloblastoma: analysis of surgical treatment carried out in 60 patients between 1977 and 1998. *Journal of Craniofacial Surgery*.

[19] Laborde A., Nicot R., Wojcik T., Ferri J., Raoul G. (2017). Ameloblastoma of the jaws: management and recurrence rate. *European Annals Otorhinolaryngology Head Neck Diseases [Internet]*.

[20] Neagu D., Escuder-de la Torre O., Vázquez-Mahía I. (2019). Surgical management of ameloblastoma. Review of literature. *Journal of Clinical and Experimental Dentistry*.

[21] Carlson E. R., Marx R. E. (2006). The ameloblastoma: primary, curative surgical management. *Journal of Oral and Maxillofacial Surgery*.

[22] Ruslin M., Hendra F. N., Vojdani A. (2017). The Epidemiology, treatment, and complication of ameloblastoma in East-Indonesia: 6 years retrospective study. *Medicina Oral, Patología Oral y Cirugía Bucal*.

[23] Kanda Y. (2013). Investigation of the freely available easy-to-use software “EZR” for medical statistics. *Bone Marrow Transplantation*.

[24] Rankow R. M., Hickey M. J. (1954). Adamantinoma of the mandible: analysis of surgical treatment. *Surgery*.

[25] Masson J. K., Mcdonald J. R., Figi F. A. (1959). Adamantinoma of the jaws; a clinicopathologic study of 100 histologically proved cases. *Plastic and Reconstructive Surgery*.

[26] Sehdev M. K., Huvos A. G., Strong E. W., Gerold F. P., Willis G. W. (1974). Proceedings: ameloblastoma of maxilla and mandible. *Cancer*.

[27] Daramola J. O., Ajagbe H. A., Oluwasanmi J. O. (1980). Recurrent ameloblastoma of the jaws--a review of 22 cases. *Plastic and Reconstructive Surgery*.

[28] Shatkin S., Hoffmeister F. S. (1965). Ameloblastoma: a rational approach to therapy. *Oral Surgery, Oral Medicine, Oral Pathology*.

[29] Müller H., Slootweg P. J. (1985). The ameloblastoma, the controversial approach to therapy. *Journal of Maxillofacial Surgery*.

[30] Ueno S., Mushimoto K., Shirasu R. (1989). Prognostic evaluation of ameloblastoma based on histologic and radiographic typing. *Journal of Oral and Maxillofacial Surgery*.

[31] Eckardt A. M., Kokemüller H., Flemming P., Schultze A. (2009). Recurrent ameloblastoma following osseous reconstruction--a review of twenty years. *Journal of Cranio-Maxillofacial Surgery*.

[32] Nakamura N., Higuchi Y., Mitsuyasu T., Sandra F., Ohishi M. (2002). Comparison of long-term results between different approaches to ameloblastoma. *Oral Surgery, Oral Medicine, Oral Pathology, Oral Radiology & Endodontics*.

[33] Antonoglou G. N., Sándor G. K. (2015). Recurrence rates of intraosseous ameloblastomas of the jaws: a systematic review of conservative versus aggressive treatment approaches and meta-Analysis of non-randomized studies. *Journal of Cranio-Maxillofacial Surgery*.

[34] Hertog D., Bloemena E., Aartman I. H. A., van-der-Waal I. (2012). Histopathology of ameloblastoma of the jaws; some critical observations based on a 40 years single institution experience. *Medicina Oral, Patología Oral y Cirugía Bucal*.

[35] Fregnani E. R., da Cruz Perez D. E., de Almeida O. P., Kowalski L. P., Soares F. A., de Abreu Alves F. (2010). Clinicopathological study and treatment outcomes of 121 cases of ameloblastomas. *International Journal of Oral Maxillofacial Surgery [Internet]*.

[36] Reichart P. A., Philipsen H. P., Sonner S. (1995). Ameloblastoma: biological profile of 3677 cases. *European Journal of Cancer: Part B—Oral Oncology*.

[37] Au S. W., Li K. Y., Choi W. S., Su Y. X. (2019). Risk factors for recurrence of ameloblastoma: a long-term follow-up retrospective study. *International Journal of Oral and Maxillofacial Surgery*.

[38] Olaitan A. A., Arole G., Adekeye E. O. (1998). Recurrent ameloblastoma of the jaws. A follow-up study. *International Journal of Oral and Maxillofacial Surgery*.

[39] Yang Y. C., Wang J. J., Huang Y., Cai W. X., Tao Q. (2021). Development and validation of a prognostic nomogram for postoperative recurrence-free survival of ameloblastoma. *Cancer Management and Research*.

[40] Fonseca F. P., Monteiro Benites B., Soares C. D. (2018). Prognostic importance of FGF2 and FGFR1 expression for patients affected by ameloblastoma. *Journal of Oral Pathology & Medicine*.

[41] Adebayo E. T., Fomete B., Adekeye E. O. (2011). Delayed soft tissue recurrence after treatment of ameloblastoma in a black African: case report and review of the literature. *Journal of Cranio-Maxillofacial Surgery*.

[42] Hayward J. R. (1973). Recurrent ameloblastoma 30 years after surgical treatment. *Journal of Oral Surgery*.

[43] Collings S. J., Harrison A. (1993). Recurrent ameloblastoma?--An historic case report and a review of the literature. *British Dental Journal*.

[44] Arotiba G. T., Effiom A. O., Ayodele A. S. O. (2012). A classification system for recurrent ameloblastoma of the jaws--review of 30 cases in Nigerians. *Nigerian Quarterly Journal of Hospital Medicine*.

[45] Grafft M. L., Sazima H. J., Parker F. P., Rappaport I. (1970). Ameloblastoma recurring in previously placed iliac crest autograft: report of case. *Journal of Oral Surgery*.

[46] Stea G. (1985). Recurrence of an ameloblastoma in an autogenous iliac bone graft. *Journal of Oral and Maxillofacial Surgery*.

[47] Vasan N. T. (1995). Recurrent ameloblastoma in an autogenous bone graft after 28 years: a case report. *New Zealand Dental Journal*.

[48] Choi Y. S., Asaumi J., Yanagi Y., Hisatomi M., Konouchi H., Kishi K. (2006). A case of recurrent ameloblastoma developing in an autogenous iliac bone graft 20 years after the initial treatment. *Dentomaxillofacial Radiology*.

